# Editorials

**Published:** 1890-09

**Authors:** 


### HOMŒOPATHIC PHYSICIAN,

A MONTHLY JOURNAL OF

HOMŒOPATHIC MATERIA MEDICA AND CLINICAL MEDICINE.

"If our school ever gives up the strict inductive method of Hahnemann, we are lost, and deserve only to be mentioned as a caricature in the history of medicine."—CONSTANTINE HERING.

Vol. X. SEPTEMBER, 1890. No. 9.

### EDITORIALS.

THE TREATMENT OF AFFECTIONS OF THE HEART, particularly in respect of exercise, has undergone decided modifications during the past few years on the part of the allopaths. When a German physician announced that he had found organic heart troubles were materially benefited by prolonged exercise, his ideas were so at variance with the prevailing treatment, which was usually enforced quiet—with drugs, of course—that the allopathic medical world was astounded.

Their usual habit of failing to individualize was the cause of this shock.

By even slight observation he who has had homœopathic training can readily see that exercise, judiciously carried out, is of vast importance in the treatment of this class of affections. Familiarizing himself, as far as possible, with the character of the lesion, he should not hesitate to impress upon the sufferer the necessity of following advice in this direction.

There is much to be done here beside finding the curative medicinal remedy.

Dietetics have a large share in making for the patient's good. Care should be exercised regarding the quantity and the quality of both solid and liquid foods.

Thus, for example, where the tendency is to fatty degeneration, fat-producing food should be avoided.

The taking of liquids, too, should be looked after with care, for it is a fact that an excess of fluid food causes great discomfort. A dry diet is of prime importance. Especially during meals should liquids be avoided, or taken only in sips. At other times but sufficient to supply the requirements of the kidneys should be taken, and what is taken should not be cold. Too much is apt to retard absorption, and this will be followed by distension of the stomach. This, again, interferes with the action of the diaphragm, and thus the heart's action is impeded. With valvular disease of the heart we usually find sluggish digestion, fermentation of food, and the formation of gas. This distending the stomach and pressing on the lungs through the diaphragm causes dyspnœa. Indeed, all the organs of digestion are liable to disturbance in this affection, and it is incumbent upon us to ever bear this in mind.

Congestion of the liver is one of the concomitants in many cases of valvular disease, owing to the condition of the circulation through the lungs, which is secondary to the heart lesion. Venous fullness of all the abdominal viscera usually follows. Hence, even a light error in diet will be followed by the greatest distress.

We are sure that only to mention these facts will be sufficient to impress even the careless observer that they are real, and that they must be given attention.

But a short time ago a case of heart affection came under observation which would have been saved much suffering had the mongrel who was treating the case known of the above facts. The patient was being given two drugs in alternation, one of which, we judged from the odor, was digitalis tincture. The patient had that peculiar sinking and feeling of goneness in the abdomen which is characteristic of digitalis, and was being fed on beef-tea and milk punch, while she was protesting all the while against taking food, for she had no appetite. Notwithstanding this she was blindly following the advice of the mongrel, and was suffering severely as a consequence.

G. H. C.

Chronic, or Interstitial Pneumonia, is defined in one of the latest old-school works as "never a primary affection, but is commonly secondary to croupous and catarrhal pneumonia from failure of resolution. It may also result from hæmorrhagic infarctions and abscess of the lungs, and is sometimes associated with tubercular deposit."

There is no affection which goes more to show the harm of mongrel and old-school treatment than so-called chronic pneumonia. Four words quoted above—"from failure of resolution"—tell nothing of the cause of that failure. To cover the entire ground and read correctly there should be added after "resolution," from too much drugging.

The history of every case of this disease which we have seen, is this: An attack of acute pneumonia. Old-school treatment, or that of a progressive (?) homœopathist. Weeks, and perhaps months, in bed. Months of cough, usually with profuse, fetid expectoration; night-sweats, hectic fever, loss of appetite, emaciation, marked dyspnœa, sleeplessness, and altogether symptoms which lead the patients and their friends to believe that consumption is present. A pitiable condition, indeed—and all due to treatment by crude drugs, and much of them!

Has any Hahnemannian, after treating a case of acute pneumonia, ever seen chronic pneumonia? Some years ago Dr. Lippe answered this question so far as his personal experience went, with a decided no. Dr. Fellger's answer was the same. And this after many years of practice.

In this affection the treatment is the same old story—Quinine and Morphia. Agents which go to suspend absorbent action and to prevent resolution. The exudation will then degenerate and complete cirrhosis follows. Many of these cases cannot be cured by even Hahnemannian Homœopathy. The constitutional condition is so overpowered that death is merely a question of time. In the meantime the patient is passing a miserable existence. And all this is due to treatment. Nature alone and unassisted would do better. It has been shown repeatedly that no treatment is better than drugging. And yet regulars continue to kill, and mongrels still assist them, and at the same time boast of progressive Homœopathy. G. H. C.

Dr. P. P. Wells.—It affords us pleasure to let Dr. Wells' numerous friends know that he has in a measure recovered from his attack of paralysis, and that he is remarkably well, with the exception of some weakness of the feet.

A recent pleasant visit to him shows his mental vigor as strong as ever, and that his vast knowledge of Hahnemannian Homœopathy carries him along, and continues to make him its strong defender.

The world owes a debt to Dr. Wells, and Hahnemannians everywhere should not forget what is due him for his energetic labors for the good of the cause. G. H. C.

---

Destroying the Bacillus of Tubercle.—At the International Medical Congress, in Berlin, on August 14th, Dr. Koch, the bacteriologist, read an address on "Bacteriology" in which we find the following: "The new points were some observations on tuberculosis, as observed in the fowl, and on the possible curative treatment of phthisis by drugs. In a series of experiments which he lately conducted, he found that certain bodies, such as volatile oils, and certain metallic salts, such as nitrate of silver, and preparations of gold, even in very small doses (1 in 1,000,000, and even less) destroy the tubercle bacilli in a very short time, and he thus believes that it is not impossible that in the course of time some drug may be found which will effectively destroy the bacillus without injuring the body."

From this are we justified in saying we believe "the world do move"? And yet how slowly even the most advanced members of the allopathic world "do move." What a happy day when, "in the course of time some drug may be found which will effectively destroy the bacillus without injuring the body!" If they could only understand the nature of disease they would not be seeking "some drug," but they would bend their energies toward knowing how better to use what drugs they now possess. Hahnemannian Homœopathy can give them this information. It is free to all in the *Organon*, the *Materia Medica Pura*, and in the works of those faithful followers of Hahnemann, who have implicitly adhered to his teachings. The line that Koch and his blind followers are clinging to is not in the true path; 'tis the path that leads but to the grave—the allopath.

